# The Best Society

**Published:** 1873-12

**Authors:** 


### THE BEST SOCIETY.

I KNOW a mother who, not long ago, walked the streets of New York all night long, with her little daughter of twelve years old, because she had no place to sleep. She was the daughter of a man who was once rich, and she herself was a deserving woman.

There are hundreds of persons every night in New York who are furnished with a plank bench to lie down on at the police offices. Many of them are peaceable, have committed no crime, but have no money to purchase a night's lodging.

If stout and strong men are found every night who must beg a night's lodging, what shall become of poor women, without work, or friend, or home, the last cent having been spent for a penny roll of bread; and what terrible temptations are they under to commit one wrong act, and then have money at command? And yet what multitudes of brave hearts are there who had rather go roofless and sleepless the bitterest night—had rather go and die, as some do, poor things! Is not that the "best society," which has been in operation three months, called

"THE FRATERNALS,"

whose object is to afford women a place to sleep, who would otherwise be obliged to walk the streets all night, or go to the police stations and sleep with outcast vagabonds and drunken men in the same room? Too true! During the three months hundreds of women have found a night's shelter from the cold and storms, and things a thousand times worse! No doubt the good Dr. Deems is at the bottom of this, but where the "Free Dormitory for Women" is we cannot say.

Men and brethren, and sisters all, everywhere, can't every one of you send a dime or two to help a cause like this to the Rev. Washington Gledden, editor of the *Independent*, No. 3 Park place, New York?

